# Portuguese cork industry: filling the knowledge gap regarding occupational exposure to fungi and related health effects

**DOI:** 10.3389/fpubh.2024.1355094

**Published:** 2024-06-04

**Authors:** Carla Viegas, Marta Dias, Cátia Pacífico, Tiago Faria, Anália Clérigo, Hermínia Brites, Liliana Aranha Caetano, Elisabete Carolino, Anita Quintal Gomes, Susana Viegas

**Affiliations:** ^1^H&TRC – Health and Technology Research Center, ESTeSL – Escola Superior de Tecnologia e Saúde, Instituto Politécnico de Lisboa, Lisbon, Portugal; ^2^NOVA National School of Public Health, Public Health Research Centre, Comprehensive Health Research Center, CHRC, REAL, CCAL, NOVA University Lisbon, Lisbon, Portugal; ^3^Centro de Ciências e Tecnologias Nucleares (C2TN), Instituto Superior Técnico, Universidade de Lisboa, Lisbon, Portugal; ^4^Research Institute for Medicines (iMed.ULisboa), Faculty of Pharmacy, University of Lisbon, Lisbon, Portugal; ^5^Instituto de Medicina Molecular, Faculdade de Medicina de Lisboa, Lisbon, Portugal

**Keywords:** *Penicillium glabrum* complex, *Aspergillus* sp., spirometry, health effects, suberosis

## Abstract

**Introduction:**

The presence of the *Penicillium* section *Aspergilloides* (formerly known as *Penicillium glabrum*) in the cork industry involves the risk of respiratory diseases such as suberosis.

**Methods:**

The aim of this study was to corroborate the predominant fungi present in this occupational environment by performing a mycological analysis of 360 workers’ nasal exudates collected by nasal swabs. Additionally, evaluation of respiratory disorders among the cork workers was also performed by spirometry.

**Results:**

*Penicillium* section *Aspergilloides* was detected by qPCR in 37 out of the 360 nasal swabs collected from workers’ samples. From those, 25 remained negative for *Penicillium* sp. when using culture-based methods. A significant association was found between ventilatory defects and years of work in the cork industry, with those people working for 10 or more years in this industry having an approximately two-fold increased risk of having ventilatory defects compared to those working less time in this setting. Among the workers who detected the presence of *Penicillium* section *Aspergilloides*, those with symptoms presented slightly higher average values of CFU.

**Discussion:**

Overall, the results obtained in this study show that working in the cork industry may have adverse effects on worker’s respiratory health. Nevertheless, more studies are needed (e.g., using serological assays) to clarify the impact of each risk factor (fungi and dust) on disease etiology.

## 1 Introduction

Portugal produced 49.6% of all worldwide cork in 2019, with 640 companies working in this production sector with 8,343 direct workers and an overall profit of 718 M euros each year (1). Additionally, two-thirds of worldwide cork exportation originates in Portugal, 77.4% from semi-processed products, 82.3% from processed products from natural cork, and 68% from agglomerate products.

The presence of the *Penicillium* section *Aspergilloides* (formerly known as *Penicillium glabrum*) in this industry involves the risk of respiratory diseases such as suberosis, a type of hypersensitivity pneumonitis that is one of the most prevalent diseases among cork workers (2–9). Epidemiologic studies have already reported an estimated prevalence between 9 and 19% of suberosis among Portuguese cork workers (3).

*Penicillium* section *Aspergilloides* and *Chrysonilia sitophila* were both reported as the dominant fungal species in all stages of cork production (10–12), corroborating their role in respiratory disorders in this setting (10, 12, 13). In addition, despite not being fully understood, an altered immune response to inhalation of antigens produced by these species can also trigger in susceptible individuals an inflammatory cascade that can progress to lung fibrosis (14).

*Aspergillus* section *Fumigati*, one of the most ubiquitous saprophytic fungi (15), has also been observed in cork industries (12). It is suggested as an indicator of harmful fungal contamination in different occupational environments (16–18), with several fungal species from the *Fumigati* section implicated in the development of suberosis (9, 19). Thus, an additional health risk should be considered for exposed workers (17, 18, 20).

*Aspergillus* section *Fumigati* is also ranked as a fungal species of critical priority, as it is considered one of the potential pathogenic species with higher clinical relevance, partly due to the prevalence of azole-resistant phenotypes both in clinical and environmental isolates (21).

A pilot study has previously shown that exposure to particles is also a concern particularly associated with the respirable fraction that occurs during manual intervention in the task of agglomerating cork (12).

The nose cavity is the primary entry point for inhaled air and, consequently, the first region of the respiratory tract in contact with airborne fungi, among other occupational risk factors (22–27). In this context, the use of the nasal swab procedure for sampling is of utmost importance since it allows fungal detection in the nasal cavity, being an easy and painless collection method that can be performed everywhere with no need for additional equipment (22, 25).

The aim of this study was to corroborate the predominant fungi present in this occupational environment by performing a mycological analysis of 360 workers’ nasal exudates collected by nasal swabs. Additionally, evaluation of respiratory impairment among the cork workers was also performed through spirometry.

## 2 Materials and methods

### 2.1 Previous environmental monitoring

**Figure 1:**
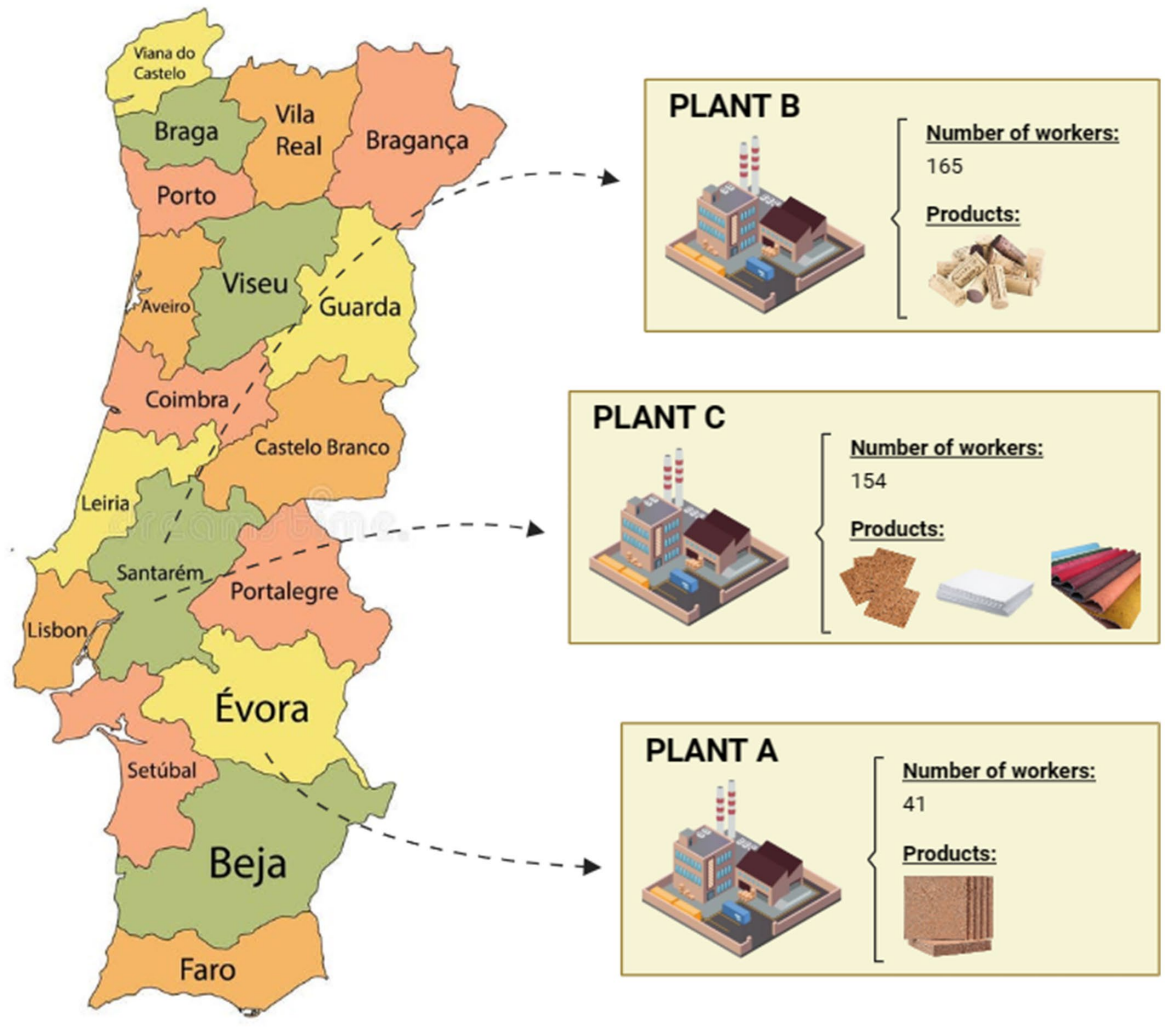
Geographical distribution of the cork plants assessed.

Three cork plants were included in the study developed between January and February 2014. Plant A was located in the Évora district, while plants B and C were located in the Santarém district. Plant A employed 41 workers and produced cork boards for further processing by other industries. Plant B employed 165 workers and mainly produced natural bottle corks. Plant C employed 154 workers and specialized in several cork-derived articles such as cork tiles, papers, and textiles (Figure 1).

All three plants provide respiratory protection equipment (RPE) to their workers, but workers do not use this equipment in a consistent manner. All the plants work 5 days a week in two 8-h shifts. To assess occupational exposure to fungal contamination, air samples of 50–100 L were collected through an impaction method with a flow rate of 140 L min-1 onto malt extract agar (MEA) supplemented with chloramphenicol (0.05%) (Frilabo, Portugal) using the Millipore air Tester (Millipore). Surface samples were collected by swabbing the surfaces of the same indoor sites, using a 10-by-10 cm square stencil disinfected with 70% alcohol solution between samples according to the International Standard ISO 18593 (2004). The obtained swabs were then plated onto MEA. Air samples of 250 L were collected using the impinger Coriolis μ air sampler (Bertin Technologies) at 300 L min-1 airflow rate. Samples were collected onto 10 mL sterile phosphate-buffered saline with 0.05% Triton X-100, and the collection liquid was subsequently used for DNA extraction. Samples collected were analyzed using culture-based (air samples collected by impaction and surface samples) and molecular methods (air samples collected by impinger) following the procedures applied in previous research work (12).

In the previous study (12), Plant C showed an increased air fungal diversity compared with the other two plants, among which the most prevalent was *Penicillium* sp. (76.5%). The distribution of fungal species in the surface samples of Plants A and B was similar, with isolates from the *Aspergillus* section *Fumigati* being the only ones found besides *C. sitophila*. In Plant C, the most prevalent genera were *Trichoderma* sp. and *Penicillium* sp. (52.9%; 29.4%). All three plants had higher fungal loads indoors than outdoors. Real-time PCR identified the *Penicillium* section *Aspergilloides* in 10 out of the 12 air samples, that is, in six more sampling sites than the culture-based methods (12).

### 2.2 Study population

In total, 360 workers from the 3 companies were enrolled in the study (plant A—41 workers, plant B—165 workers, and plant C—154 workers). A control group (38) with administrative tasks outside these companies was also engaged in the study. The 360 workers from the three cork plants participated in both the nasal swab assay and spirometric study.

All workers and control group subjects gave written informed consent to participate in the study. This study complied with the Helsinki Declaration and Oviedo Convention, and all data were stored and analyzed in accordance with the Portuguese General Data Protection Regulation (GDPR) law n° 58/2019.

### 2.3 Nasal swab assay

Two consecutive swab samples, with sterilized cotton swabs, were taken from one nostril at the end of the work shift. The swabs were rotated against the internal anterior walls of the nostril and then placed in the provided transport tube. One of the swab samples of each worker was plated onto malt extract agar (MEA) supplemented with chloramphenicol (0.05%) (Frilabo, Portugal). The samples collected this way were subsequently incubated at 27°C for 5 to 7 days. The fungal species were quantified (CFU per worker) and identified microscopically through macro and microscopic characteristics according to De Hoog et al. (28).

The other swab sample was eluted into 1 mL of PBS, centrifuged at 250 rpm (5 g) for 30 s, and then frozen at −80°C until DNA extraction. This sample was subsequently centrifuged for 30 min at 3500 rpm. The supernatant was discarded, and the pellet was re-suspended in 200 μL of distilled water. DNA was then extracted using the ZR Fungal/Bacterial DNA MiniPrep Kit (Zymo Research, United States) according to the recommendations of the manufacturer. Molecular identification of *Penicillium* section *Aspergilloides* (*P. glabrum* complex) and *Aspergillus* section *Fumigati* (Table 1) was achieved by real-time PCR (RT-PCR) using the Rotor-Gene 6,000 qPCR Detection System (Corbett-Quiagen, Germany). Primers and probes for *Penicillium* section *Aspergelloides* were designed with Primer Express software for the Calmodulin (CaM) gene of *Penicillium* section *Aspergilloides* strain AS3.15335. Primers for *Aspergillus* section *Fumigati* were described by Cruz-Perez et al. (29). Reactions included 1× iQ Supermix (Bio-Rad, Portugal), 0.5 μM of each primer (Table 1), and 0.375 μM of TaqMan probe in a total volume of 20 μL. Amplification followed a three-step PCR: 40 cycles with denaturation at 95°C for 30 s, annealing at 52°C for 30 s, and extension at 72°C for 30 s. For each gene amplified, a non-template control and a positive control were included. The positive control consisted of DNA obtained from a reference strain belonging to the culture collection of the Reference Unit for Parasitic and Fungal Infections, Department of Infectious Diseases of the National Institute of Health Dr. Ricardo Jorge was included. These strains have been sequenced for ITS, B-tubulin, and calmodulin.

**Table 1:** Sequence of primers and TaqMan probes used for real-time PCR.

Fungal species targeted	Sequences	Reference
**Penicillium section *Aspergilloides***		
Primer forward	5‘-TGCCTGGACCGGAACCTA-3′	
Primer reverse	5‘-CACCATCGCCATCCTTGTC-3‘	Designed for this study
Probe	5‘-TGAATGCTTTCCCGTAATA-3′	(information above)
***Aspergillus* section *Fumigati***		
Primer Forward	5‘-CGCGTCCGGTCCTCG-3‘	
Primer Reverse	5‘-TTAGAAAAATAAAGTTGGGTGTCGG −3′	(29)
Probe	5‘-TGTCACCTGCTCTGTAGGCCCG −3′	

### 2.4 Spirometry

An individual questionnaire was applied to obtain data on (1) smoking habits, (2) history of known lung disease, (3) presence of respiratory symptoms, and (4) exposure history.

Spirometries were performed using an MK8 Microlab spirometer. The spirometer was always calibrated before data collection, with a 3-L syringe to a total of 12 L. Values from calibration were accepted if results were within a ± 3% range. The spirometer used met the international standards with respect to flow rate and duration of the test. A minimum of three acceptable flow-volume curves were obtained, and repeatability was verified on the two tests with the largest forced vital capacity (FVC) and forced expiratory volume in 1 s (FEV1), according to ATS/ERS 2005 guidelines (30). The following respiratory function parameters were evaluated: FVC, FEV1, and FEV1/FVC%.

A control group was not considered since the aim of our study was to identify the prevalence rate of ventilatory defects in exposed workers through comparison with reference values from the European Community for Coal and Steel (ECCS) (31). Taking this into consideration, the methodology normally used in lung function laboratories was considered suitable for this study. For interpretation purposes, the fixed cutoff of 80% of the predicted value was used. Ventilatory defects were classified as follows: (1) obstructive—FEV1/FVC% below 80%; (2) restrictive—FEV1 and FVC below 80% with a FEV1/FVC% equal or above 80%; and (3) non-specific—FEV1, FVC, and FEV1/FVC% below 80%.

### 2.5 Statistical analyses

Statistical analysis of all data was performed using the Statistical Package for Social Sciences (SPSS) version 24.0 for Windows. To characterize the workers’ samples quantitatively, frequency analysis (*n*, %) for qualitative data and calculation of minimum, maximum, mean, and standard deviation were used. The criterion for significance was set at *p* < 0.05. The Shapiro–Wilk test was used to test the normality of the quantitative data. To study the association between two qualitative variables, the Chi-Square Test was used to determine whether the applicability assumptions were verified or Fisher’s Exact Test otherwise. Binary logistic regression was used to identify risk factors for the presence of respiratory symptoms. Once the assumption of normality was verified, the t-test was used to compare the presence of *Penicillium* section *Aspergilloides* between those workers who have and those who do not have respiratory symptoms.

## 3 Results

### 3.1 Nasal swab assay

#### 3.1.1 Culture-based methods

**Figure 2:**
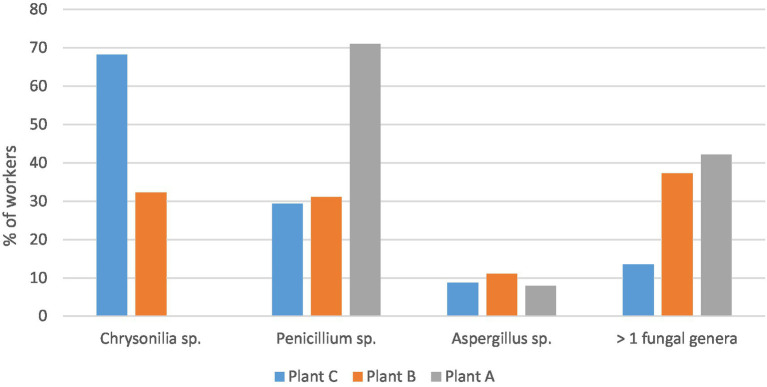
Fungal contamination distribution in the workers’ nasal swabs.

Among the 360 workers subject to nasal swab assay, 310 (86.1%) presented fungal contamination. In 119 workers, overgrowth of *Chrysonilia sitophila* was observed, which rendered impossible the quantification of the number of isolates on the plate, being considered in these cases 500 isolates per nostril, following previous procedures regarding environmental samples´ fungal quantification (12). Around 36.6% of the workers’ nasal swabs presented *Penicillium* genus, 9.9% *Aspergillus* sp., and 29.1% observed more than one fungal genera (Figure 2). Within the 38 samples from the control group, 16 (42.1%) did not show any fungal growth, 44.7% presented *Penicillium* sp., and 18.4% *Cladosporium* sp. The sample from one subject presented *Mucor* sp. and other *Geotrichum* sp.

**Figure 3:**
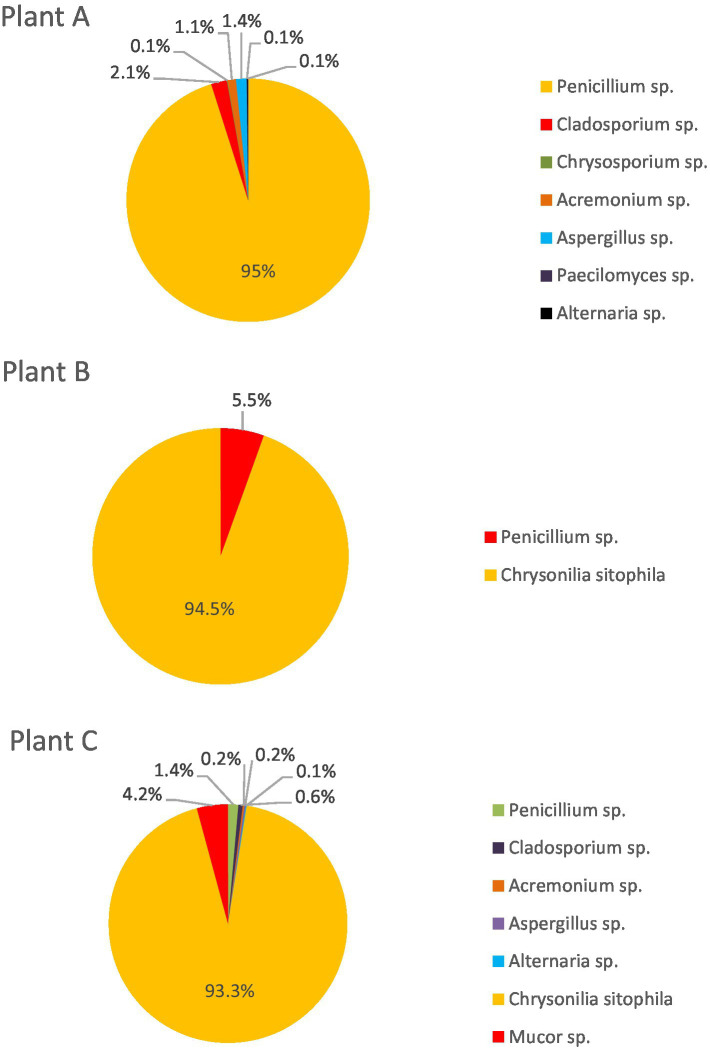
Fungal distribution in the workers’ nasal swab assays in the 3 cork plants.

Considering the 500 isolates per nostril on the plates where overgrowth was observed, *C. sytophila* (92.3%) was the most common fungi found in the workers’ noses, followed by *Penicillium* sp. (4.9%), *Rhizopus* sp. (1.5%), and *Mucor* sp. (0.7%). *Cladosporium* sp., *Alternaria* sp., *Acremonium* sp., and *Aspergillus* sp. were present in lower counts, accounting for the majority of the remaining percentage. When considering workers from each cork plant, *C. sitophila* was the most common fungus isolated in workers from both plants B and C, accounting for more than 90% of the fungal diversity, while plant A presented a slightly different fungal distribution. *Penicillium* sp. represented 95.0% of the fungal species identified, followed by *Cladosporium* sp. (2.1%), *Aspergillus* sp. (1.4%), and *Acremonium* sp. (1.1%). *Alternaria* sp., *Paecilomyces* sp., and *Chrysosporium* sp. accounted for 0.1% each (Figure 3).

Fungal diversity is described in Table 2 according to the isolates number obtained in the workers’ noses from the 3 plants.

**Table 2:** Fungal isolates distribution in workers’ noses from the 3 cork plants.

Isolates/worker nose.	Genera/Species
0–100	*Chrysosporium* sp.
	*Acremonium* sp.
	*Alternaria* sp.
	*Scopulariopsis* sp.
	*Fusarium verticilloides*
	*Aureobasidium* sp.
	*Neoscytalidium hialinum*
	*Neoscytalidium dimiatum*
	*Geomyces* sp.
	*Geotrichum* sp.
	*Fusarium poae*
	*Fusarium oxysporum*
	*Cladophialophora* sp.
	*Aspergillus* sp.
100–500	*Cladosporium* sp.
500–1,000	*Mucor* sp.
1,000–2,500	*Rhizopus* sp.
> 2,500	*Chrysonilia sitophila*
	*Penicillium* sp.

#### 3.1.2 Molecular tools

**Figure 4:**
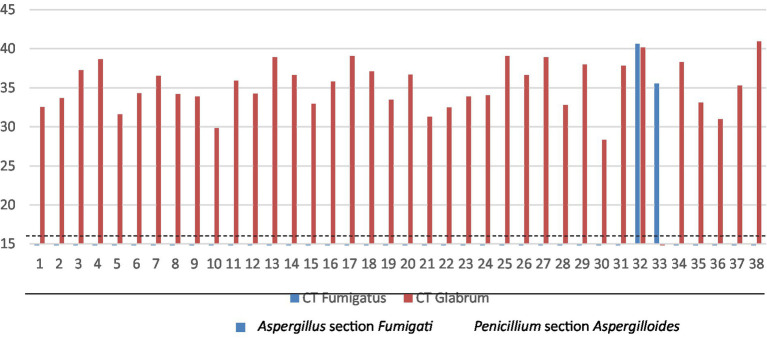
CT values of each sample for *Penicillium* section *Aspergilloides* and *Aspergillus* section *Fumigati*. The dashed line represents the CT of the positive control for *Penicillium* section *Aspergilloides* while the filled line represents the CT of the positive control for *Aspergillus* section *Fumigati*.

We next subjected the nasal swab samples from the 360 workers of the three different plants (Plant A—41; Plant B—165; Plant C—154) to qPCR analysis and observed successful amplification of DNA from *Penicillium* section *Aspergilloides* in 37 of the analyzed samples. From those, 25 remained negative for *Penicillium* sp. when using culture-based methods. Furthermore, in one worker*, Aspergillus* section *Fumigati* was co-amplified with *Penicillium section Aspergilloides,* and in another worker, that section was detected singularly. As expected, in the 38 controls used, none were positive for the *Penicillium section Aspergilloides* nor for the *Aspergillus* section *Fumigati.* Of note, samples with lower cycle threshold (CT) values very likely exhibited higher levels of the detected fungi (Figure 4).

### 3.2 Spirometry

Three hundred sixty workers completed the symptom questionnaire and performed spirometry. Since 40 workers had previous pulmonary pathology, only 320 were considered in the analysis. The average age of participants was 41.24 ± 10.56, and 66.9% (*n* = 214) were men. A considerable percentage (*n* = 118; 36.9%) of participants were smokers (Table 3).

**Table 3:** General characteristics of the workers’ samples.

Characteristics		*n* (%)	Minimum – Maximum	Mean ± Std. deviation
Age (years)			18–65	41.24 ± 10.56
Gender	Male	214 (66.9%)		
	Female	106 (33.1%)		
Height (cm)			148–198	169.97 ± 8.81
Weight (kg)			38–136	73.42 ± 13.66
Number of years in cork industry			0–51	11.02 ± 8.86
	<10 years	152 (47.5%)		
	≥10 years	168 (52.5%)		
Smoking habits	No	177 (55.3%)		
	Yes	118 (36.9%)		
	Ex-smoker^a^	25 (7.8%)		

^a^An adult who has smoked at least 100 cigarettes in his or her lifetime but who had quit smoking at the time of the interview.

The average number of years of work in the cork industry was 11.02 ± 8.86. The majority (*n* = 168, 52.5%) worked in this industry for 10 or more years and did not smoke (*n* = 177, 55.3%). Regarding the respiratory symptoms, the majority (*n* = 193, 61.3%) did not have symptoms (Table 4).

**Table 4:** Spirometry data of cork workers according to smoking habits and exposure.

Smoker	Spirometry	Number of years in cork industry (exposure)									
		<10					≥10				
		*n*	Min	Max	Mean	SD	*N*	Min	Max	Mean	SD
No	FEV_1_% Predictive	68	77.00	137.00	100.01	11.05	109	72.00	141.00	100.82	12.89
	FVC % Predictive	68	79.00	136.00	101.90	11.00	109	72.00	231.00	106.61	17.43
	FEV_1_/FVC % baseline	68	58.00	96.00	82.56	6.76	109	62.00	97.00	80.92	5.88
Yes	FEV_1_% Predictive	73	74.00	118.00	97.41	10.89	45	63.00	142.00	96.96	16.58
	FVC % Predictive	73	79.00	126.00	101.22	11.73	45	66.00	138.00	100.96	15.21
	FEV_1_/FVC % baseline	73	60.00	96.00	80.66	6.45	45	65.00	92.00	78.82	6.61
Ex-smoker	FEV_1_% Predictive	11	67.00	114.00	94.09	15.57	14	80.00	116.00	95.07	11.95
	FVC % Predictive	11	73.00	115.00	94.82	14.48	14	77.00	114.00	95.93	10.21
	FEV_1_/FVC % baseline	11	74.00	87.00	82.27	3.88	14	68.00	88.00	80.43	6.28

Min, Minimum; Max, Maximum; SD, Standard Deviation.

Concerning the ventilatory defects, 36.5% of spirometries (*n* = 115) were classified as obstructive, 0.6% (*n* = 2) as restrictive, and 1.6% (*n* = 5) as non-specific. A significant association was found between smoking habits and age with ventilatory defects ( $\chi_{1}^{2}$ = 5.376, *p* = 0.020 and $\chi_{1}^{2}$ = 31.565, *p* < 0.001, respectively). For each additional year of life, the risk of the presence of ventilatory defects increased [Odds Ratio = 1.788, Confidence Interval_95%_ = (1.420, 2.252)], and in the smokers or ex-smokers, the risk of ventilatory defects was approximately 2 times higher [Odds Ratio = 1.734, Confidence Interval_95%_ = (1.087, 2.768)] (Tables 5, 6).

**Table 5:** Ventilatory defects versus smoking habits.

		Smoker		Chi-Square test		
		No smoker	Smoker	Test statistic	df	*p*-vlaue
Ventilatory defects	Absence	131/198 (66.2%)	62/117 (53.0%)	5,376a	1	,020*
	Presence^a^	67/198 (33.8%)	55/117 (47.0%)			

^a^Presence corresponds to Obstructive or Restrictiveor Mixed; *Significant association at a 5% significance level.

**Table 6:** Ventilatory defects versus age.

		Age					Chi-Square test		
		<25	[25; 35]	[35; 45]	[45; 55]	≥ 55	Test statistic	df	*p*-vlaue
Ventilatory defects	Absence	14/17 (82.4%)	58/81 (71.6%)	64/87 (73.6%)	46/96 (47.9%)	11/34 (32.4%)	31,565^a^	4	,000
	Presence^a^	3/17 (17.6%)	23/81 (28.4%)	23/87 (26.4%)	50/96 (52.1%)	23/34 (67.6%)			

^a^Presence corresponds to Obstructive + Restrictive + Mixed; *Significant association at a 5% significance level.

A significant association was found between ventilatory defects and years of work in the cork industry ( $\chi_{1}^{2}$ = 5.058, *p* = 0.025), and it was found that those who worked for 10 or more years in this industry had an approximately two-fold increased risk of having ventilatory defects [Odds Ratio = 1.692, Confidence Interval_95%_ = (1.068, 2.681)], in relation to those who have worked for less than 10 years. Regarding respiratory symptoms, namely regular cough, expectoration, wheezing, and dyspnea, no significant association was detected with the number of years of work in this industry (Table 7).

**Table 7:** Ventilatory defects and respiratory symptoms among exposed workers.

		Number of years in cork industry (exposure)		Qui-square test		
		<10 Count/column total (%)	≥ 10 Count/column total (%)	Test statistic	df	*p*-value
Ventilatory defects	Absence	101/149 (67.8%)	92/166 (55.4%)	5.058	1	0.025*
	Presence	48/149 (32.2%)	74/166 (44.6%)			
Cough regularly?	No	132/152 (86.8%)	138/168 (82.1%)	1.337	1	0.248
	Yes	20/152 (13.2%)	30/168 (17.9%)			
Do you have expectoration, regularly?	No	138/152 (90.8%)	149/168 (88.7%)	0.380	1	0.538
	Yes	14/152 (9.2%)	19/168 (11.3%)			
Do you have wheezing regularly?	No	152/152 (100%)	166/168 (98.8%)			0.500^b^
	Yes	0/152 (0%)	2/168 (1.2%)			
Do you have dyspnea regularly?	No	152/152 (100%)	168/168 (100%)			
	Yes	0/152 (0%)	0/168 (0%)			

^a^Changed corresponds to Obstructive + Restrictive + Mixed; ^b^ Fisher Exact Test; *Significant association at a 5% significance level.

The same analysis was performed separately in smokers and non-smokers. In non-smoking workers, a significant association was found between ventilatory defects and years of exposure ( $\chi_{1}^{2}$ = 5.762, *p* = 0.016). It was found that those people who worked for 10 years or more in the cork industry had a two-fold increased risk of developing respiratory defects [Odds Ratio = 2.002, Confidence Interval_95%_ = (1.131, 3.543)], in relation to those who have worked for less than 10 years. In smokers, no significant association was found between the number of years of exposure and ventilatory defects ( $\chi_{1}^{2}$ = 0.586, *p* = 0.444). However, although not significant, a 1.4-time higher risk of developing ventilatory defects [Odds Ratio = 1.411, Confidence Interval_95%_ = (0.582, 3.419)] was found in smokers who worked for 10 or more years in the cork industry (Table 8).

**Table 8:** Ventilatory defects and respiratory symptoms among exposed workers in non-smokers and smokers.

			Number of years in cork industry (exposure)		Chi-square test		
			<10 Count/column total (%)	> = 10 Count/column total (%)	Test Statistic	df	*p*-value
No Smoker	Ventilatory defects	Absence	64/89 (71.9%)	78/139 (56.1%)	5.762	1	0.016*
		Presence^a^	25/89 (28.1%)	61/139 (43.9%)			
	Do you have a persistent cough?	No	83/91 (91.2%)	125/142 (88.0%)	0.586	1	0.444
		Yes	8/91 (8.8%)	17/142 (12.0%)			
	Do you have expetoration?	No	83/91 (91.2%)	132/142 (93.0%)	0.238	1	0.626
		Yes	8/91 (8.8%)	10/142 (7.0%)			
	Do you have wheezing regularly?	No	90/91 (98.9%)	137/142 (96.5%)			0.408^b^
		Yes	1/91 (1.1%)	5/142 (3.5%)			
	Do you have dyspnea?	No	91/91 (100%)	139/142 (97.9%)			0.283^b^
		Yes	0/91 (0%)	3/142 (1.3%)			
Smoker	Ventilatory defects	Absence	44/78 (56.4%)	19/48 (39.6%)	3.365	1	0.067
		Presence^a^	34/78 (43.6%)	29/48 (60.4%)			
	Do you have a persistent cough?	No	73/79 (92.4%)	41/48 (85.4%)			0.237^b^
		Yes	6/79 (7.6%)	7/48 (14.6%)			
	Do you have expetoration?	No	69/79 (87.3%)	38/48 (79.2%)	1.504	1	0.220
		Yes	10/79 (12.7%)	10/48 (2.8%)			
	Do you have wheezing regularly?	No	78/79 (98.7%)	48/48 (100%)			1.000^b^
		Yes	1/79 (1.3%)	0/48 (0%)			
	Do you have dyspnea?	No	78/79 (98.7%)	47/48 (97.9%)			1.000^b^
		Yes	1/79 (1.3%)	1/48 (2.1%)			

^a^Presence corresponds to Obstructive + Restrictive + Mixed; ^b^Fisher Exact Test; *Significant association at a 5% significance level.

In smokers who did not use RPE devices, a significant association was detected between respiratory defects and the number of years of exposure ( $\chi_{1}^{2}$ = 5.399, *p* = 0.020), and it was found that workers who were in for 10 or more years in the cork industry presented a two-fold higher risk of developing respiratory defects [Odds Ratio = 2.190, Confidence Interval_95%_ = (1.124, 4.270)]. In non-smokers who used RPE, no significant association was detected ( $\chi_{1}^{2}$ = 0.213, *p* = 0.644). Regarding smokers who did not use an individual respiratory protection device, a significant association between the number of years of exposure and ventilatory defects was found ( $\chi_{1}^{2}$ = 4.356, *p* = 0.037). Those who have been working for 10 or more years in the cork industry had a two-fold increased risk of developing respiratory defects [Odds Ratio = 2.269, Confidence Interval_95%_ = (1.045, 4.928)]. Finally, for workers who smoked in the past and who used RPE, no significant association between respiratory defects and years of exposure was found ( $\chi_{1}^{2}$ = 0.034, *p* = 0.853) (Table 9).

**Table 9:** Smoking habits versus always use RPE versus number of years of exposure.

			Number of years in cork industry (exposure)		Chi-square test		
			<10	≥ 10	Test Statistic	df	*p*-value
Non-smoker and does not always use individual protection	Ventilatory defects	Absence	48/66 (72.7%)	56/102 (54.9%)	5.399^b^	1	0.020*
		Presence^a^	18/66 (27.3%)	46/102 (45.1%)			
Non-smoker and always uses individual protection	Ventilatory defects	Absence	12/18 (66.7%)	18/30 (60.0%)	.213^b^	1	0.644
		Presence^a^	6/18 (33.3%)	12/30 (40.0%)			
Smoker and does not always use individual protection	Ventilatory defects	Absence	40/68 (58.8%)	17/44 (38.6%)	4.356^b^	1	0.037*
		Presence^a^	28/48 (41.2%)	27/44 (61.4%)			
Smoker and always uses individual protection	Ventilatory defects	Absence	4/9 (44.4%)	2/4 (50.0%)			1.000^b^
		Presence^a^	5/9 (55.6%)	2/4 (50.0%)			

^a^Presence corresponds to Obstructive or Restrictive or Mixed; ^b^Fisher Exact Test; *Significant association at a 5% significance level.

No significant association was found between the presence of *Penicillium* section *Aspergilloides* and *Aspergillus* section *Fumigati* in nasal swabs with respiratory symptoms (*p* = 1.000 and $\chi_{1}^{2}$ = 0.007, *p* = 0.934, respectively, Table 10). However, it was observed that in the absence of both fungal species, the majority of workers did not have any respiratory symptoms (99.5 and 89.6%, respectively) (Table 10).

**Table 10:** Prevalence of respiratory symptoms according to the presence of *Penicillium* section *Aspergilloides* and *Aspergillus* section *Fumigati* (qPCR results).

			Ventilatory defects		Qui-Square test		
			Absence	Presence	Test Statistic	df	*p*-value
*Aspergillus* section *Fumigati*	Absence	count/row total (%)	192/193 (99.5%)	121/122 (99.2%)			1.000^a^
	Presence	count/row total (%)	1/193 (0.5%)	1/122 (0.8%)			
*Penicillium* section *Aspergilloides*	Absence	count/row total (%)	173/193 (89.6%)	109/122 (89.3%)	0.007	1	0.934
	Presence	count/row total (%)	20/193 (10.4%)	13/122 (10.7%)			

^a^Fisher Exact Test.

Considering only the cases in which the presence of *Penicillium* section *Aspergilloides* was detected, no statistically significant differences were observed between those workers who did not have respiratory symptoms and those who had (t_30_ = −0.791, *p* = 0.435). However, it was verified that those workers who had symptoms presented slightly higher average values of CFU (Mean_No symptoms_ = 34.75 ± 2.87, Mean_With symptoms_ = 35.61 ± 3.23).

## 4 Discussion

### 4.1 Main findings

This study found a significant association between ventilatory defects and years of work in the cork industry. Indeed, the risk of having ventilatory defects was approximately two-fold higher in workers who had worked for 10 or more years compared to those who had worked for less than 10 years in this industry. The same trend was observed in smokers who did not use respiratory protective equipment and worked for 10 years or more. Although *Penicillium* section *Aspergilloides* was detected in workers´ noses, the study did not find any association between respiratory effects and fungal contamination.

### 4.2 Nasal fungal contamination

*Chrysonilia sitophila* was the most prevalent fungi on workers’ noses, according to previous environmental monitoring at the same plants (12). Higher fungal diversity was observed by workers’ nose sampling [compared to environmental sampling obtained in the previous study (12)] and in Plant A (compared to the other two plants). In workers from plant A, *Penicillium* sp. was the most prevalent fungal genus, whereas, in workers from the other two plants, *C. sitophila* was dominant. The number of workers and type of activities inside the facilities appears to influence fungal contamination (32–34). In fact, Plant A had fewer workers and produced only cork boards, while Plants B and C produced more cork-derived articles [natural bottle corks, cork tiles, papers, and textiles (12)]. The organic dust contamination present in the cork industry is critical for workers’ exposure since particles act as carriers of fungi to the upper airways (34–36). Fungal geographic distribution and dominance also vary with climate-driven patterns (37, 38), which explains the differences observed in Plant A, located in south Portugal, with warmer average temperatures.

In this study, qPCR enabled the detection of *Penicillium* section *Aspergilloides* in 25 samples where *Penicillium* sp. had not been identified by culture. On the other hand, in highly contaminated environments, fast-growing species such as *C. sitophila* can inhibit the growth of *Penicillium* and *Aspergillus* sp. in culture (33, 34, 39, 40). Furthermore, molecular detection can be underestimated due to PCR inhibitors such as environmental contaminants (e.g., dust) (41, 42). Importantly, for occupational exposure assessments, it is crucial to determine the viability of microorganisms as it relates to inflammatory and cytotoxic effects (34, 40, 43–46). Altogether, this evidence highlights the relevance of combining culture-based methods with molecular detection (30, 47, 48).

Although workers in whom *Penicillium* section *Aspergilloides* was detected showed slightly higher values of symptoms, the association between this contaminant and respiratory disorders (10, 13) was not significant in this study. *Aspergillus* section *Fumigati*, on the other hand, was detected in two workers and can explain their reported symptoms. *Aspergillus* section *Fumigati* is commonly related to respiratory symptoms due to the small size of the conidia and to other virulence factors. Allergic bronchopulmonary aspergillosis (ABPA), rhinitis, rhinosinusitis, and severe asthma with fungal sensitization (SAFS) are some of the diseases more often associated with occupational exposure to *Aspergillus* genera (49, 50).

Whereas *Aspergillus* section *Fumigati* is critical for its public health burden and urgent need for surveillance, Mucorales and *Fusarium* spp. are also prioritized due to limited therapeutic options and fungal cross-resistance to azoles used in agriculture (21). The agricultural use of azole fungicides has been linked to the emergence of antifungal resistance in clinical practice (51). To prevent fungal infections of cork oaks, azole fungicides such as tebuconazole and benzimidazole have been used (52, 53), making the cork production sector a hotspot for the development of azole resistance. To prevent antifungal resistance, it is crucial to raise awareness and adopt interlinked, integrated, and innovative multisectoral approaches to surveillance in occupational exposure assessments.

### 4.3 Spirometry

Considering lung function evaluation, both smokers and non-smokers with longer exposure showed a higher prevalence rate of ventilatory defects.

We observed a significant association between ventilatory defects and years of work in the cork industry. In fact, those people who worked for 10 or more years in this industry had an approximately two-fold increased risk of having ventilatory defects. This is of particular relevance to demonstrate causality between working in the cork industry (prone to organic dust and fungi) and ventilatory defects and agrees with results previously published (6, 7, 54, 55). The average number of years of workers in the cork industry analyzed in this study was relatively small (11.02 ± 8.86 years). As such, a further increase in the years of exposure (only 52% had more than 10 years of exposure) might have an important effect on worker’s health. However, the “healthy worker effect” (HWE) needs to be considered, given that severely ill and chronically disabled are commonly excluded from employment (56), leading to lower overall death rates or morbidity when compared with the general population. Other occupational epidemiologists simply describe HWE as the reduction of mortality or morbidity of occupational cohorts when compared with the general population (57). It is a special form of selection bias common to occupational cohort studies previously noted in populations occupationally exposed to different risk factors (36, 58). This might imply that more workers have health effects but already left the company at the moment of the study, resulting in the employed workforce having fewer sick people than expected. Moreover, the ventilatory defects observed in cork industry workers engaged in this study can be due to the combination of different risk factors present in the cork industry, such as cork dust and fungal contamination (54). Previous studies noted that occupational exposure could present higher health impacts among workers than smoking and that both exposures resulted in worse outcomes (59). Furthermore, long-term exposure in susceptible individuals may lead to lung fibrosis and, therefore, a restrictive ventilatory defect (60). Thus, smokers who have already had an airway disease and the related obstructive ventilatory defect are also expected to have a restrictive defect.

## 5 Conclusion

Our study showed that working in the cork industry may have adverse effects on worker’s respiratory health. Even using a relatively low exposure-time window, it was possible to detect health effects in workers, evidencing the need to invest in risk management measures that can eliminate or reduce exposure to fungi and dust in this setting. Cork industry workplaces normally have high contamination of both fungi and dust. Thus, preventing exposure to organic dust also prevents exposure to fungi. Therefore, process containment and enclosure and, if not achievable, adequate ventilation systems (general mechanical ventilation and proper local exhaust ventilation) should be implemented. If these options are not possible to implement, then respiratory protection devices must be chosen and available as the only protection measures, particularly in tasks that involve manual handling of cork. Nevertheless, more studies are needed (e.g., using serological assays) to clarify the impact of each risk factor (fungi and dust) on disease etiology.

## Data availability statement

The original contributions presented in the study are included in the article/supplementary material, further inquiries can be directed to the corresponding author/s.

## Ethics statement

The studies were conducted in accordance with the local legislation and institutional requirements. The participants provided their written informed consent to participate in this study.

## Author contributions

CV: Conceptualization, Formal analysis, Funding acquisition, Investigation, Methodology, Project administration, Resources, Writing – original draft, Writing – review & editing. MD: Formal analysis, Writing – original draft. CP: Formal analysis, Writing – original draft. TF: Formal analysis, Writing – original draft. AC: Formal analysis, Resources, Writing – original draft. HB: Formal analysis, Resources, Writing – original draft. LC: Formal analysis, Writing – original draft. EC: Formal analysis, Writing – original draft. AG: Formal analysis, Writing – original draft. SV: Investigation, Methodology, Resources, Writing – original draft, Writing – review & editing.
